# Selected Predictors of the Sense of Intimacy in Relationships of Young Adults

**DOI:** 10.3390/ijerph16224447

**Published:** 2019-11-13

**Authors:** Dorota Czyżowska, Ewa Gurba, Natalia Czyżowska, Alicja Kalus, Katarzyna Sitnik-Warchulska, Bernadetta Izydorczyk

**Affiliations:** 1 Institute of Psychology, Faculty of Philosophy, Jagiellonian University in Krakow, 30-060 Krakow, Poland; ewa.gurba@uj.edu.pl; 2 Institute of Psychology, Pedagogical University in Krakow, 30-084 Krakow, Poland; natalia.czyzowska@up.krakow.pl; 3 Institute of Psychology, University of Opole, 45-052 Opole, Poland; akalus@uni.opole.pl; 4 Institute of Applied Psychology, Faculty of Management and Social Communication, Jagiellonian University in Krakow, 30-348 Krakow, Poland; k.sitnikwarchulska@gmail.com (K.S.-W.); bernadetta.izydorczyk@uj.edu.pl (B.I.)

**Keywords:** intimacy, closeness, engagement, communication, satisfaction in relationships

## Abstract

The main research objective of this study was seeking the predictive role of closeness to parents, attachment, identity style, identity commitment, type of relationship, and having children in intimacy among young women and men. Many studies indicate differences in the level of engagement, communication, and satisfaction in relationships. The study group comprised 227 people, including 114 women (M = 29.99; SD = 4.36), and 113 men (M = 30.00; SD = 4.33). A total of 40% of the subjects were married, and the remaining 60% subjects were in informal relationships; 101 people had children and the other individuals were childless. The following instruments were used: The Miller Social Intimacy Scale, questionnaires to assess closeness and attachment, and the Identity Style Inventory. The significance of the differences and the stepwise regression analysis were performed. The results of the study demonstrated a higher level of intimacy in a relationship with a partner among women than men. The nature of a relationship does not matter to the sense of intimacy. However, closeness to parents during childhood and adolescence, the model of interpersonal relations, and the identity style are predictors of intimacy in a relationship. The study results can be used in creating preventive and educational programs focused on family life and satisfied relationships.

## 1. Introduction

Intimacy is a multidimensional and ambiguous construct. Muniruzzaman [1] indicates that the meaning of intimacy, that denotes a close interpersonal relationship, varies between and within relationships. 

In the literature, there are different understandings of this term depending on the context in which it appears [2]. Intimacy is sometimes considered in the context of sexuality, and then understood as a sexual bond but it is also viewed more broadly, as a tendency to discover oneself in relationships with others [3,4]. Most often, however, it appears as a property of the dyadic relationship [3]. The interpersonal nature of intimacy and individual differences in ability and motivation to intimacy are emphasized by Plopa [5], who associates it with a disposition to care for each other, to open oneself to the other person, and to enjoy the closeness, as well as with empathy. The key intimacy features include love, tenderness, trust, and opening up to the other person [2,6]. Showing our inner world to the partner and entrusting him with secrets is possible when one trusts the partner and believes that they will not be revealed, that he or she will not reveal intimate details of our lives [7]. By exposing oneself to another person, revealing the emotions, usually, a symmetrical reaction from the entrusted person is expected [8].

Intimacy can be of varying degrees of intensity, and its character depends both on the nature of a relationship and the stage of acquaintance. Researchers of this phenomenon indicate the importance of three of its elements: opening up, the ability to accept the partner’s perspective and experiencing positive emotions in a relationship [6,9]. Intimacy is a property of long-term relationships in which partners strive to maintain them. It is a feature of developing relationships between people who share experiences, communicate their own thoughts, feelings, and views with each other. It manifests itself both verbally and non-verbally and involves many spheres of life [10,11].

An understanding of intimacy as one of the three factors of love, apart from passion and engagement is proposed by Robert Sternberg [12]. In his view, intimacy means a sense of support in a relationship and a sense of closeness between the partners [12]. The clusters of intimacy, according to the author, include caring for the well-being of a loved one, sharing the possessed goods, the option to count on a partner’s help, experiencing happiness in contact with him or her, as well as giving and receiving emotional support.

Intimacy as a result of a positive solution to a crisis in the period of early adulthood is considered in the theory of psychosocial development by Erikson [13]. According to the author, a young individual who established his or her own identity should possess the “ability to develop true and mutual psychological intimacy with another person” ([13], pp. 90–91). A negative solution to the crisis leads to isolation resulting in difficulties in building close relationships, including romantic relationships, which has an impact on the further development of the individual. According to Erikson [13], the ability to develop intimacy fosters creativity, productivity, and ego integration. 

Intimacy is an important characteristic of close relationships and largely determines the quality of these relationships, as well as the satisfaction with intimacy for the partners [14,15,16,17,18]. It is important for both women and men, although there are some gender differences in this area [19,20]. Women display more need to open up to a partner and share their experiences than men. In the studies in which it was attempted to determine the relationship quality predictors, it was found that the intimacy experienced in a relationship was more important for women than men and determined satisfaction with the relationship to a greater extent [17].

Recognizing that intimacy, understood as the ability of partners to enter into a close relationship, build a sense of closeness, dependence, and support, is a significant characteristic of romantic relationships and can state the degree of satisfaction with a relationship, an attempt to determine the factors conditioning its intensity was undertaken. It is worth noting that although intimacy is often considered as an essential component of love or an important characteristic of an intimate relationship, it is much less frequent that researchers attempt to identify factors that may be relevant to its building and experience. Therefore, in the presented author’s studies, it was checked which of the specified variables played a role in experiencing intimacy in romantic relationships of young people. 

It is worth mentioning that the relation between attachment with parents during childhood and adolescence and experiencing intimacy in adulthood has so far been mainly brought into theoretical concepts. There is a lack of studies that measure the full range of conditions for intimacy. Moreover, there are no Polish studies related to psychosocial predictors of intimacy in emerging adulthood.

The article will present research aimed at determining the predictors of intimacy in a relationship with a partner. The studied variables were defined based on both the concept of attachment and the concept of socialization. According to the authors, both approaches are complementary, not separate. Based on the theory of Erikson [13] pointing to the importance of a method of solving earlier crises for solving of the intimacy–isolation crisis, as well as on the results of empirical research, the following variables were analyzed as predictors of intimacy: closeness to parents experienced in childhood and adolescence, the way of building their own identity and attachment to close relatives when entering adulthood. Apart from the variables defining previous experiences of the person, which may be important for building the pattern of social relations, as well as defining the individual’s readiness to create a close intimate relationship, the factors related to a current life situation of the individual were taken into account, including the type of relationship (marriage vs. informal relationship) and having or not having children, and the gender of the respondents. Researchers note differences in the functioning of marital and informal relationships [21]. The results of many studies indicate differences in the level of engagement [22], communication [23,24], satisfaction with the relationship [23,25,26] in informal and marital relationships. Having children also affects the quality of relationships between spouses and partners, as well as their satisfaction with the relationship [27].

## 2. Research Objectives and Questions

The research discussed in this article is part of a larger research project aimed at identifying the determinants of entering into lasting romantic relationships and being relevant to the quality of these relationships, as well as to the partners’ satisfaction. The objective of the presented research was to determine the dependency between closeness to parents, identity style, attachment in adulthood, and the sense of intimacy with the partner. Based on the Erikson model [13], it was assumed that earlier developmental crises and experiences from childhood and adolescence, which are reflected in the sense of closeness to parents, in the way of building identity, and in the attachment that an individual manifests in adulthood, can influence the level of intimacy experienced in a romantic relationship. Based on the findings of Hazan and Shaver [28] and the results of empirical research [18,29,30,31], it is assumed that the attachment manifested by an adult remains in connection with the style of attachment shaped during childhood, reflecting the models of interpersonal relations formed at that time [32]. Hazan and Shaver [28] believe that the relationship between partners of a romantic relationship significantly reflects the relationship of the child with his or her mother. Empirical studies indicate similarity between the style of attachment presented during childhood and adulthood [29] and provide the basis for concluding that the attachment of adults and infants has the same hidden structure and dynamics [31,33]. The meaning of gender and the type of relationship (marriage vs. informal relationship) for the studied relationships was explored as well. It was also intended to check whether the discussed dependencies between variables are of the same nature for women and men, and whether the same variables are predictors of the intimacy they experience. 

It was assumed that closeness to parents during childhood and adolescence and a safe style of attachment (low anxiety and high, safe independence) would favor a higher intimacy. These expectations were formulated on the basis of theories pointing to the importance of early experiences in shaping subjective resources of the individual [29,34] defining the ability to establish close relations and personal openness, as well as on the basis of the theory of Erikson [13] indicating the importance of early social experiences and the manner of solving earlier crises for solving subsequent crises. Similarly, the theory of Erikson, which indicates the importance of a positive solution of the identity crisis for the ability to enter into close intimate relationships and results of empirical research shows that a mature identity (informative and normative style and high engagement) will contribute to building intimacy within a relationship with a partner [35,36]. 

Erikson also assumed that in women, in contrast to men, the final solution of the identity-development crisis may take place as a result of entering into a close relationship and solving another crisis, which is the intimacy-isolation crisis. The question arises whether gender will be a predictor of intimacy, and whether the discussed variables will have the same meaning for the sense of intimacy in women and men. An additional reason for consideration about the meaning of gender for the sense of intimacy in a relationship is data indicating that women have a greater need to open up to a partner, confide in, and share their experiences, than men. Research, in which the factors assessing the quality of a relationship were determined, suggests that intimacy experienced in a relationship with the partner is more important for women than for men [17,37]. Considering differences noted in the motives of building and functioning of formal and informal relationships [21,38], the nature of a relationship was taken into account as a predictor of intimacy. It is difficult to define unequivocal expectations as to the importance of the nature of a relationship for the sense of intimacy. On one hand, it can be assumed that the marital relationship, providing a greater sense of stability and security, may be more conducive to experiencing intimacy, on the other hand, intimacy may be more intense in informal relationships, because low intimacy would lead to the breakup of the relationship. It is known that in informal relationships, the breakup can be easier [39].

Based on the results of research showing changes in the way the relationship functions and in the relations between partners as a result of the appearance of children [27,40] a model was tested in which the intimacy predictor has involved having or not having children.

The purpose of the author’s study was to determine the predictors of intimacy experienced in a relationship. Both factors referring to the previous experiences of an individual in relation to relatives, the way of constructing his or her own identity, as well as the characteristics of the current relationship that are important for the sense of intimacy in a close relationship, were taken into account. There were analyzed relations with relatives during childhood, adolescence, and entering adulthood, (closeness to parents experienced during childhood and adolescence, attachment to relatives) and the method of building one’s own identity (styles of identity), as well as factors related to the current life situation of an individual: the type of relationship (marriage vs. informal relationship) and having or not having children. 

Within the research model, the closeness to the mother/father was determined by the supportive behavior of the parent. The attachment to relatives was verified through the measure the level of anxiety and safe independence. The authors referred to Berzonsky’s concept to define the style of identity. Creating a model for the development of personal identity, Berzonsky [41] distinguishes three styles of identity (informative, normative, and diffuse–avoidant), which present different social-cognitive strategies of information processing and problem-solving. Identity styles mean a way of getting an individual to make decisions, especially those that are important to his or her identity and way of life [42]. The informative style, considered to be the most adaptive, means criticism, self-reflection, and an active search and evaluation of information that is important for the individual. People of the informative style have a high need to know, openness to new ideas, values, and activities [42]. The main characteristic of the normative style is conformism and imitation. People of this style display inflexibility in thinking, dogmatic commitment, a stable concept of “I”, and a reluctance to explore [43]. The diffuse–avoidant style means delay, evasive action, postponing decision-making, and living life from moment to moment [42]. This style is associated with a low sense of engagement and an unstable concept of “I” [43]. Also, the meaning of gender was taken into consideration. 

Research questions:

1) Is gender, type of relationship (non-formal marriage), or having or not having children important for the perceived level of intimacy in a relationship with a partner?
2) Which of the considered variables: closeness to the mother; closeness to the father; attachment (level of safe independence and fear of rejection); identity style (normative, informative, diffuse–avoidant); identity commitment, type of relationship; having children, are predictors of the level of intimacy in a relationship?
3) Are there differences in conditioning the experienced level of intimacy in a close relationship between young women and men?

## 3. Material and Methods

### 3.1. Procedure

The research was conducted individually, the participation was voluntary and people did not receive any gratifications for taking part. The respondents were informed that they could resign from participation in the study at any time. In order to provide the respondents with as much anonymity as possible, they were only asked to verbally give their consent to the participation in the study. All people agreed. Students of the Institute of Psychology at the Jagiellonian University participated in the collection of research material. The non-probability sampling was used. The respondents who were met individually at a place convenient for them were sequentially completing the questionnaires in order to assess the level of intimacy, closeness to parents, attachment, identity style, and metrics prepared for the study. 

### 3.2. Characteristics of the Study Group 

When selecting the group of respondents, the following criteria were determined: 20+ years of age, being in an intimate relationship for a period of at least 12 months. Those who were in an intimate relationship at the time of the study, but the relationship lasted for less than a year, were excluded from the study. 

The study group consisted of 227 people aged 23 to 36 participated in the study (M = 30; SD = 4.35), including 114 women (50.2%) (M = 29.99; SD = 4.36), and 113 men (49.8%) (M = 30.00; SD = 4.33). All the individuals were in relationships for at least a year, of which 40% were married and the remaining 60% were in informal relationships. People had secondary and higher education and came from the country, as well as small and large cities. Some people had children and the other individuals were childless. The characteristics of the group are presented in Table 1.

### 3.3. Instruments

To measure the level of intimacy the Miller Social Intimacy Scale (MSIS) was used [44]. The scale measures both the affective and the cognitive aspects of intimacy. It consists of 17 questions to which a person responds based on a 10-point scale. In the case of 11 questions (e.g., “How satisfying is the relationship with her/with him?”), a respondent determines the intensity on a scale from “not really” to “a bit” to “much”. In the case of 6 questions, a respondent determines the frequency (e.g., “How often do you feel closeness to him/her?”) based on a scale from “very rarely” through “sometimes” to “almost always”. The reliability of a scale is measured by the Cronbach’s α coefficient in research by the author of the method was satisfactory and varied from 0.86 to 0.91. The reliability of the scale measured by a test–retest was r = 0.91 for a sample surveyed at a two-month interval and r = 0.84 for a sample surveyed at a one-month interval [44]. In the presented study, the Cronbach’s α coefficient was 0.96. 

Closeness to parents was determined based on an adapted questionnaire prepared by Marek Regnerus [45], “Closeness to biological mother and father”. A respondent is asked to answer based on a 5-point scale (1 means never, and 5 means always) to 6 questions regarding the relationship with mother and father (e.g., “Could you count on help in problem situations?”). When completing the questionnaire, the individual has to refer to the period of childhood and adolescence. In his research, Regnerus (2012) obtained high reliability of the questionnaire: on the scale of closeness to mother α = 0.89, and on the scale of closeness to father α = 0.92. In the survey analyzed by this paper, these were as follows: α = 0.87 and α = 0.98, accordingly.

Attachment to close relatives was assessed on the basis of a questionnaire prepared by Regnerus [45]. The questionnaire consists of 12 statements about relationships with close relatives (e.g., “I find it difficult to allow myself to rely on others.”, “I know I will find people where I need them.”), 6 of which is a scale of safe independence and the other 6, a scale of anxiety. The task of a respondent is to determine the extent to which particular statements characterize him or her based on a 5-point scale (from 1—“does not characterize me at all” to 5—“characterizes me fully”). A high score on the scale of safe independence and low on the scale of anxiety can be treated as a measure of safe attachment. In the research conducted by Regnerus [45], the reliability of the questionnaire was satisfactory and, for the scale of independence, α = 0.80, and for the scale of anxiety α = 0.82. In the study survey, there were also obtained satisfactory indicators: the scale of independence—α = 0.96, the scale of anxiety—α = 0.72. 

The identity was analyzed using the Identity Style Inventory (ISI-3) by Michael Berzonsky in the Polish adaptation of Alicja Senejko [46]. The questionnaire allows determining the level of engagement and three types of identity styles: informative, normative, and diffuse–avoidant. The questionnaire consists of 40 statements describing beliefs, attitudes and behaviors, to which an individual responds on a 5-point scale, determining to what extent particular statements characterize him or her. The questionnaire allows determining the level of engagement and the intensity of particular identity styles. The reliability of the questionnaire calculated using Cronbach’s α coefficient on a sample of Polish adolescents was: for the informative style from 0.61 to 0.74, for the normative style from 0.58 to 0.74, for the diffuse–avoidant style from 0.68 to 0.81, and for engagement from 0.62 to 0.78 [46]. Similar indicators were obtained in the survey analyzed by this paper.

### 3.4. Ethical Approval

This study was determined to conform to the 1964 Helsinki declaration and its 215 later amendments of comparable ethical standards and was conducted in accordance with national regulations and guidelines. The written informed consent was obtained from all participants. The protocol was approved by the Committee for Ethics of Scientific Research at the Institute of Psychology, Jagiellonian University, Krakow.

### 3.5. Statistical Methods

The study results were statistically analyzed using the IBM SPSS Statistics (Statistical Package for the Social Sciences) software. In the first stage of statistical analysis, the significance of differences between the average values of intimacy intensity level was measured. In the second stage, a forward stepwise regression analysis was performed. An attempt was made to estimate the strength and direction of the influence of possible psychological predictors of the sense of intimacy, which could be verified in the study model. Due to a large number of explanatory variables, a forward stepwise regression was applied, in which a successive inclusion of relevant predictors to the model was made, until the appearance of the first predictors, whose level of significance exceeded the permissible values of *p* < 0.05. In this way, the models were purged of unnecessary and weak predictors, and only models that had satisfactory prediction remained.

As an acceptance or rejection criterion for the proposed hypotheses, the level of significance was set at *p* = 0.05. 

## 4. Results

In order to answer the question about differences in the sense of intimacy in a relationship with the partner between women and men, a Student’s t-test was carried out (Table 2), which indicated differences in this respect. Women declared a higher level of intimacy than men. On the other hand, there were no differences in the level of intimacy between people in marital relationships and informal relationships. An analysis of the level of intimacy in the group of people having children and not having children indicated a higher level of experienced intimacy in childless relationships.

In order to determine predictors of the sense of intimacy, a multiple stepwise regression analysis was performed, where the dependent variable was the level of experienced intimacy, and the following were considered as the predictors: gender, closeness to mother and father, attachment, whose measures include fear of rejection and safe independence, and identity styles (informative, normative and diffuse–avoidant), and engagement (Table 3). There were variables included that reflect the individual’s previous experience, especially in relation to relationships with close relatives or are a result of the way of solving previous tasks or developmental crises. The predictors in the final model explain a total of 12.6% of the variance of the dependent variable.

Due to the fact that gender turned out to differentiate the level of intimacy and constitute its predictor, it was decided to conduct separate analyses for women and men in order to determine if the same factors are important for their sense of intimacy. As the predictors of intimacy, the following were taken into account: type of relationship, having or not having children, closeness to mother and father, fear of rejection, and safe independence as a measure of attachment, identity styles (informative, normative, and diffuse–avoidant), as well as identity involvement. The predictors in the final model explain a total of 17% of the variance of the dependent variable in the case of women and a little over 9% in the case of men. 

## 5. Discussion

The aim of the research was to determine the factors relevant to the level of experiencing intimacy in a romantic relationship during early adulthood. It was assumed that the intimacy taken in a relationship with the partner is an important element of the quality and permanence of a romantic relationship. Based on Erikson’s theory [13], it was also assumed that the ability to be in a close relationship and to experience intimacy is an expression of a positive solution to earlier crises, in particular to the identity crisis. 

The obtained results indicate that the studied women experience a higher level of intimacy than studied men. Our research results are consistent with the results of Beam et al. [37]. Here, it is worth mentioning the observational studies conducted by Fishman [47], which indicated that women display greater interest than their partners’ interest in interacting with the partner, and more often show behaviors at its initiation and maintenance. Moreover, in other studies, the authors noticed a relationship between expectations for relatives, including a romantic partner, and readiness for deeper intimacy, showing care, and trust towards a partner as well [48,49].

The conducted research indicated that the nature of a relationship does not matter to the experience of intimacy in the studied group. Therefore, regardless of whether the relationship is formal or is not formalized, people may experience a similar level of intimacy. There are grounds to suppose that having children may not be meaningless for the sense of intimacy in a relationship. Conducted analyses may suggest that having children may slightly reduce the sense of intimacy. However, it should be noted that this variable did not prove to be a predictor of intimacy. Having children turned out to be irrelevant to the level of experienced intimacy when all the variables (sex, type of relationship, closeness to mother and father, attachment, identity styles, and engagement) were introduced to the model. The final model of the regression analysis indicated that both gender and closeness to mother are important for the experienced level of intimacy; the fear of rejection is treated as a measure of attachment, and the informative style. It turned out, then, that the individual’s previous experience, including relations with parents and a developed model of interpersonal relations found in the attachment style, as well as the way of building one’s identity, determine the individual’s ability to experience intimacy in a relationship. 

The obtained results indicating the importance of relations with parents for building a close relationship and intimacy with a partner are consistent with results of other research proving interdependencies between experiences in the family of origin and interpersonal competences revealing themselves in romantic and family relationships [50,51]. Also, the result showing the importance of fear of rejection, being one of the dimensions of attachment, for the level of intimacy, is consistent with results of studies showing a relation between attachment and the quality of relationship with a partner (e.g., [52,53,54,55,56]), as well as intimacy as one of the measures of love indicated by Stenberg [57]. Weisskirch [18] also indicated a similar relation. He found that identity achievement, a lack of avoidant attachment, and self-efficacy in romantic relationships are predictors of intimacy. However, the study involved different groups of subjects—postgraduate students. 

The obtained result is also consistent with the results of studies showing a relation between the level of anxiety experienced by partners and the way they function in a relationship [58], as well as the engagement in the relationship [59]. The relation between the identity style and the sense of intimacy is not surprising. The research of this paper shows that the greater intensity of the informative style favors the sense of intimacy in a relationship. The significance of identity for achieving intimacy was indicated, among others, in studies by Montgomery [35], as well as Beyers and Seiffge-Krenke [36]. Also, previous studies allowed us to conclude that people who entered adulthood were characterized by higher identity statuses (identity achievement or foreclosure) are more advanced in the development of intimacy in heterosexual relationships [60]. Moreover, results of research involving the meaning of a mature concept of identity for the strength of love in early adulthood [61] could be mentioned here. Assuming that the informative style is an equivalent of a mature concept of identity and intimacy is a component of love [62], the results obtained in the research of the paper may confirm the conclusions formulated earlier. The informative style, which means openness to new experiences and readiness to transform one’s own “I”, may favor openness to the other person, but also expressing and sharing one’s own experiences with the partner.

Due to the fact that one of the predictors of intimacy turned out to be gender and the analysis comparing the level of intimacy felt by women and men indicated differences in this area, which allows presuming that gender is an important variable in the context of intimacy, it was decided to conduct separate analyses for women and men. Their aim was to check whether the same variables were significant for intimacy experienced by women and men in a relationship. Analyses indicated that in women, the following are important: closeness to mother, anxiety that is one of the dimensions of attachment, and the informational identity style. As noted above, these results confirm the previously determined dependencies. For intimacy experienced by men in a relationship with a female partner, it was only engagement that Berzonsky [63,64] recognizes as an important characteristic of identity processes. Engagement defines the sense of objectives set by an individual and gives direction to his or her action. It refers to the clarity of standards, objectives, and beliefs that he or she has, and remains in relation to the informative and normative styles. Treating engagement as an important characteristic of identity processes, it can be concluded that the result obtained in the research of the paper confirms results of studies showing the relation between intimacy and identity. The results of the presented research may also suggest that identity engagement defining the clarity of standards and beliefs adopted by an individual translates into a more conscious entering into a relationship and engagement in its construction, which may contribute to a deeper sense of intimacy.

The study of the paper undoubtedly allowed a better understanding of the conditions of the sense of intimacy in a close relationship. It confirmed, to a certain extent, the assumptions of Erikson’s theory [13], but also attachment theories [28,32], as well as the results of previous research showing the importance of experiences in relationships with relatives during childhood and adolescence, including, in particular, parents, and the importance of a created model of interpersonal relationships, as well as of a manner of resolving the identity crisis, for the ability to enter into a relationship and experience intimacy. It is worth noting that factors related to experiences from earlier stages of an individual’s life and directly related to the way of resolving developmental crises and coping with developmental tasks turned out to be more important than factors related to the current situation of partners, which, in the research of the paper, was determined by the nature of the current relationship of the respondents (marriage or informal relationship) and the fact of having children.

We live in times when, on one hand, young people postpone the moment of entering into lasting intimate relationships or even give up such relationships by choosing to live alone, on the other hand, relationships that were intended to be lasting more and more often break up. In this context, it seems important to indicate factors determining the ability of young adults to build relationships, as well as factors determining the durability and quality of these relationships. There is no doubt that one of the important characteristics of a long-term relationship is intimacy, which is important both for the quality of a relationship and its duration. The research aimed at understanding the conditions for building a lasting and satisfying relationship is not only of a cognitive value, but can also help in the creation of educational programs preparing for family life. The prevention, taking into account the specificity of emerging Polish adults and aimed at supporting development of identity and effective relationships seems to be valuable. In the light of the results of the research of the paper, there may be a phenomenon of the signalization of modern, Polish people in emerging adulthood as an effect of not only economic and socio-cultural changes in industrialized and globalized societies, but also a lack of closeness in previous relations with parents and experiencing fear in current interpersonal relations, as well as postponing building mature identity by young adults. These factors, in fact, lower the level of experienced intimacy and, consequently, may constitute an obstacle to establishing and deepening relationships based on intimacy.

### Limitations and Future Directions

It should not be forgotten that the research of the paper included only a few variables that could be predictors of intimacy, which, in total, explain over a dozen percent of the variance of the dependent variable. This means that it is worth to carry out research that will allow indicating further factors, e.g., the characteristics of a partner and his or her engagement in the relationship, which are important for intimacy. In the future, it would be beneficial to take into account more detailed characteristics of a relationship, and in the case of having children, also their number and age. It can be expected that a relationship between spouses or partners may look slightly different depending on the age of the child, as well as the duration of marriage and parenthood. The theoretical research model assumed the potential impact of many predictors, including sociodemographic and psychological variables. As a consequence, the R-squared values were small but significant. Noting the limitations of the research of the paper, the relatively small number of respondents should be taken into account. Initially, a larger group of subjects was assumed, but some completed questionnaires proved impossible to analyze. However, these were individual, paper–pencil studies in emerging adults. In this context, the size of the examined group is comparable to that reported in other studies (see [18]). Future research including other variables may reveal other determinants of intimacy, not yet discovered in the literature. Valuable information may also be provided by longitudinal studies, which draw attention to the stability of the relationship between the examined variables over time. Moreover, studies including the comparisons between people of different ages could be valuable. According to authors of this article, the presented limitations do not negate the usefulness of the obtained results.

## 6. Conclusions

The analyses of the data enabled us to refer to the research questions asked by the authors. Based on them, the following conclusions can be made:

1. Women experience a higher level of intimacy in a relationship with a partner than men.
2. The nature of a relationship does not matter to the sense of intimacy among Polish emerging adults. Regardless of whether it is a marriage or a relationship that is not formalized, Polish people in emerging adulthood experience a similar level of intimacy.
3. Closeness to parents during childhood and adolescence, as well as a developed model of interpersonal relations reflected in the attachment style, and the way of constructing one’s own identity (identity style) are predictors of experiencing intimacy in a relationship among Polish emerging adults.
4. There are grounds to suppose that having children can be important for the sense of intimacy in a relationship, slightly lowering its level. However, it should be noted that this variable did not prove to be a predictor of intimacy.

## Figures and Tables

**Table 1:** Characteristics of the group (N = 227).

Participants	
Woman (114) 50.2%	Man (113) 49.8%
Married (91) 40%	Informal relationship (136) 60%
With children (101) 44.5%	Childless (126) 55.5%
Higher education (136) 60%	Secondary education (91) 40%

**Table 2:** Differences in the sense of intimacy.

**Woman**		**Man**			
M	SD	M	SD	t	p
141.2	16.22	135.71	15.88	2.56	0.01
**Marital relationships**		**Informal relationships**			
M	SD	M	SD	t	p
137.7	18.54	138.9	14.6	0.54	0.58
**With children**		**Childless**			
M	SD	M	SD	t	p
135.70	19.26	140.45	13.53	2.15	0.003

**Table 3:** Predictors explaining the level of intimacy in a relationship.

Dependent Variable	Independent Variables	
**Intimacy**	R^2^ = 0.15; F(4,207) = 9.22; *p* < 0.001 Predictors: Gender B = 0.20* Anxiety B = -0.18* Closeness to mother B = 0.15* Informative identity style B = 0.17*	
	Women	Men
	R^2^ = 0.18; F(3,103) = 7.51; *p* < 0.001 Predictors: Anxiety B = -0.21* Closeness to mother B = 0.26* Informative identity style B = 0.21*	R^2^ = 0.10; F(1,103) = 10.60; *p* < 0.001 Predictors: Engagement B = 0.31**

in- insignificant; *p < 0.05.
